# Intra-Venous Saline Infusion in Delirium Tremens

**Published:** 1901-04-13

**Authors:** 


### INTRA-VENOUS SALINE INFUSION IN DELIRIUM TREMENS.

That delirium tremens is a form of toxæmia one can hardly doubt, and Dr. Warbasse,[1] of New York, proposes to treat it, as other forms of toxæmia have been treated, by the intra-venous injection of saline solution; in other words by washing out the poison. He says that the disease is characterised by auto-intoxication. A chronic degeneration of the glandular structures of the body results from the prolonged use of alcohol, with the result that when the organism suffers from the shock of a traumatic injury the secreting and excreting structures fail, and a toxæmia results. The identity of the peculiar toxins of delirium tremens has not, however, as yet been determined, but, whatever they are, the disease results from the action on the nervous system of toxins, which by virtue of the pathological conditions present fail to become eliminated, absorbed, or neutralised. In the majority of the cases the natural resources of the organism are sufficient to overcome the toxæmia. Sometimes, however, the intoxication is fatal, even in the presence of kidneys which are able to excrete an adequate amount of urea. By saline injection one increases the amount of the circulating medium in which the toxic materials are dissolved, thereby diluting the poison and bathing the nerve centres with a more attenuated solution of the same. This increase in the amount of the circulating medium also causes increased excretion of fluids, thereby carrying off much of the poison, and in other ways improves the position of the patient. Dr. Warbasse relates a very striking case showing the benefit which resulted from the infusion of 40 oz. of saline solution at a temperature of 116° Fahr. into the median cephalic vein.

[1] New York Med. News.

